# Prevalence and Associated Factors of Musculoskeletal Disorders among Young Dentists in Indonesia

**DOI:** 10.5704/MOJ.1607.001

**Published:** 2016-07

**Authors:** P Phedy, L Gatam

**Affiliations:** Premier Bintaro Hospital, Bintaro, Indonesia

**Keywords:** Musculoskeletal disorder, dentist, neck pain, back pain, occupational hazard

## Abstract

**Introduction:** Musculoskeletal problems are often work related. Dentists have been reported to have a high prevalence of musculoskeletal problems. Dentists have to perform repetitive tasks, often in awkward and nonergonomic positions in their practice.

**Materials and Methods:** This is a cross-sectional study. Five-hundred copies of Nordic Musculoskeletal Questionnaire were distributed to dentists who participated in a congress of a regional branch of the Indonesian Dentist Association. Data such as sex, length of practice, the presence of assistance, smoking, occupational stress, body mass index, hand dominance, and exercise were collected. Dentist who had practised for more than five years were excluded.

**Results:** Two hundred and forty-one respondents fulfilled the research criteria. Musculoskeletal symptoms occurred in 63.5% respondents. Fatigue and pain were the most common manifestations of musculoskeletal symptoms among dentists (36.5 and 24.9% respectively). Prolonged sitting was the most common aggravating factor (26.6%) while exercise successfully relieved symptoms in 35.3% of respondents. Neck, upper back and lower back were the most common sites involved with prevalence of 25.7, 22.4, and 20.7% respectively. Neck was also the most common site of the symptoms preventing normal work during the preceding 12 months (8.3%). Exercise and stress are associated with the presence of musculoskeletal symptoms (p=0.01 and p<0.01 respectively). Exercise is associated with fatigue (p<0.01) and click (p<0.01), stress is associated with pain (p=0.00), stiffness (p=0.00), fatigue (p<0.01), and discomfort (p<0.01).

**Conclusions:** The prevalence of musculoskeletal disorders in young dentists is 63.5%. Neck is the most common affected region. Stress and exercise are the main associated factor for musculoskeletal problems in dentists.

## Introduction

Musculoskeletal problems are often work related^1^. Work related musculoskeletal problems occur even in health care provider. Nurses, physicians, surgeons and dentists are all prone to musculoskeletal problems^2,3^. Among health care providers, dentists have been reported to have the highest prevalence of musculoskeletal problems.

Prevalence of musculoskeletal pain among dentists reached 61%. More than 60% dentists had problem in more than one site^3^. Dentists have to perform repetitive tasks, often in awkward and non-ergonomic position during their practice. It is therefore understandable that musculoskeletal problem is almost inevitable for dentists^4^. Musculoskeletal problems afflict dental professionals even since early training years^5^.

Since musculoskeletal problem is almost inevitable for dentist,^4^ we tried to evaluate its prevalence among young dentists. We selected young dentist because we wanted to identify whether the musculoskeletal problems had started early in their career. We believed that as they were starting on a new career, they probably tended to work too enthusiastically ignoring the ergonomics. In this study, we report the prevalence of musculoskeletal problems and associated factors in young dentists who practised for less than five years in Indonesia.

## Materials and Methods

This is a cross sectional study conducted in September 2014 after obtaining informed consent prior to commencement of study. The sample of this study was selected consecutively from dentists who participated in a congress of the regional branch of the Indonesian Dentist Association in Jakarta, Indonesia. During the congress, 500 copies of Nordic Musculoskeletal Questionnaire^6^ were distributed and dentists were asked to fill in the questionnaire by themselves. The Nordic Musculoskeletal Questionnaire was developed by Nordic Council of Ministers for comparison of low back, neck, shoulder and general complaints in an epidemiological study. It comprises 40 forced-choice items identifying areas of the body causing musculoskeletal problems and 25 forced-choice questions relating to each area requiring further details on relevant issue such as incidence of accidents, functional impact, duration of problem and assessment by health care professionals. Musculoskeletal problems evaluated in this study were pain, stiffness, fatigue, discomfort, click, and neurological symptoms. “Click” was defined as clicking sound arising from any joint. Data regarding demographics, practice custom including hand dominance, and other associated factors such as exercise were also obtained. Dentist who had been practising dentistry for more than five years, or those who answered the questions incompletely, were excluded from the study. The association between the symptoms and other data (gender, length of practice, the presence of assistance, smoking, stress, body mass index) were obtained and analysed using Chi-square test. Data were analysed using SPSS version 21 software. Statistical significance was noted to be established when p value was <0.05.

## Result

Out of a total of 500 copies of the questionnaire distributed to the participants, 327 were returned. However, only 241 fulfilled the research criteria. The characteristics of the respondents are shown in Table I. All the respondents were general dentists. Mean duration of practice was 2.9±1.6 years. The body mass index ranged from 15.1 to 27 (mean 21.7±3.3). None of the dentist smoked and all were right hand dominant. Out of the 241 respondents, 63.5% had experienced musculoskeletal symptoms. Fatigue and pain were the most common manifestations of musculoskeletal symptoms among dentists. Prolonged sitting was the most common aggravating factor while exercise after work successfully relieved symptoms in 35.3% of respondents.

**Table I:** Characteristic of the respondents

Characteristics	N (%)
Sex	
Male	35 (14.5)
Female	206 (85.5)
Age (year)	
≤ 30	207 (85.9)
> 30	34 (14.1)
Working duration (year)	
≤ 2	100 (41.5)
>2	141 (58.5)
Body mass index	
≤ 23	120 (49.8)
>23	121 (50.2)
Exercise	
Sometimes	175 (72.6)
Never	66 (27.4)
Stress	34 (14.1)
Rest during working	
None	49 (20.3)
Once	156 (64.7)
Every single patient	36 (14.9)
Work with assistant	169 (70.1)
Working position	
Sitting	187 (77.6)
Standing	18 (7.5)
Alternating	36 (14.9)
Musculoskeletal symptoms	153 (63.5)
Symptoms	
Pain	60 (24.9)
Stiffness	8 (3.3)
Fatigue	88 (36.5)
Discomfort	36 (14.9)
Click	4 (1.7)
Neurological symptoms	0 (0)
Aggravating factor	
Prolonged sitting	64 (26.6)
Incorrect posture	44 (18.3)
Rotation	8 (3.3)
Lifting	0 (0)
Driving	8 (3.3)
Trauma	0 (0)
Alleviating factor	
Correct posture	48 (18.9)
Work pause	58 (24.1)
Exercise	85 (35.3)
Analgesic	41 (17)
Sitting	18 (7.5)
Bracing	18 (7.5)
Rest	123 (51)

Table II shows the proportion of symptomatic subjects according to the body regions evaluated by the Nordic Musculoskeletal Questionnaire. Neck, shoulder, upper back and lower back were the most common sites involved. Neck symptoms were was also the most common preventing normal work during the preceding 12 months.

**Table II:** Proportion of symptomatic subjects for body regions evaluated by Nordic Musculoskeletal Questionnaire

Body region	Trouble during last 12 months (%)	Problem preventing from doing normal work during last 12 months (%)	Trouble during last 7 days (%)
Neck	62 (25.7)	20 (8.3)	28 (11.6)
One or both shoulders	60 (24.9)	12 (5.0)	20 (8.3)
One or both elbows	8 (3.3)	0 (0.0)	0 (0.0)
One of both wrists/hands	32 (13.3)	8 (3.3)	8 (3.3)
Upper back	54 (22.4)	4 (1.7)	12 (5.0)
Lower back	50 (20.7)	16 (6.6)	24 (10.0)
One or both hips/thighs	16 (6.6)	0 (0.0)	0 (0.0)
One or both knees	33 (13.7)	0 (0.0)	0 (0.0)
One or both ankles/feet	41 (17)	0 (0.0)	0 (0.0)

Table III shows that only exercise and stress are associated with the presence of musculoskeletal symptoms. Exercise is associated with fatigue and click, stress is associated with pain, stiffness, fatigue, and discomfort.

**Table III:** Factors associated with musculoskeletal symptoms, pain, stiffness, fatigue, discomfort, and click

	Musculoskeletal symptoms			Pain			Stiffness			Fatigue			Discomfort			Click		
	No	Yes	p															
Sex																		
Male	13	22	0.54	27	8	0.47	35	0	0.28	21	14	0.39	27	8	0.12	35	202	0.53
Female	75	131		154	52		198	8		132	74		178	28		202	4	
Age																		
≤30	≥75	132	0.48	155	52	0.52	199	8	0.29	132	75	0.48	171	36	<0.01	203	4	0.54
30	13	21		26	8		34	0		21	13		34	0		34	0	
Body mass index																		
≤23	>40	80	0.19	92	28	0.34	112	8	<0.01	65	55	<0.01	108	12	0.02	116	4	0.06
> 23	48	73		89	32		121	0		88	33		97	24		121	0	
Exercise																		
Sometimes	72	103	0.01	127	48	0.09	167	8	0.07	120	55	<0.01	151	24	0.25	175	0	<0.01
Never	16	50		54	12		66	0		33	33		54	12		62	4	
Stress																		
Yes	83	124	<0.01	147	60	0	207	0	0	139	68	<0.01	171	36	<0.01	203	4	0.54
No	5	29		34	0		26	8		14	20		34	0		34	0	
Rest during working																		
None	17	32	0.84	41	8	0.23	41	8	0	33	16	0.09	41	8	0	49	0	0.33
Once	59	97		112	44		156	0		92	64		141	12		152	4	
Every single patient	12	24		181	8		36	0		28	8		20	16		36	0	
Work with assistant																		
Yes	67	102	0.08	125	44	0.33	161	8	0.06	107	62	0.52	141	28	0.19	165	4	0.24
No	21	51		56	16		72	0		46	26		64	8		72	0	
Working position																		
Sitting	64	123	0.19	151	36	<0.01	179	8	0.3	105	82	0	159	28	0.1	183	4	0.56
Standing	6	12		10	8		18	0		14	4		18	0		18	0	
Alternating	18	18		20	16		36	0		34	2		28	8		36	0	
Duration of practice (year)																		
Two or less	31	69	0.09	72	28	0.22	92	8	0.01	56	44	0.03	96	4	0	96	4	0.03
More than two	57	84		109	32		141	0		97	44		109	32		141	0	

## Discussions

In the present study we evaluated the prevalence of musculoskeletal symptoms in dentists. We also evaluated the factors associated with the symptoms.

The prevalence of musculoskeletal symptoms in our study is slightly higher than those reported by Chikte *et al* in a meta-analysis (53.9%)^7^. Although the prevalence in our study still fell within the 95% confidence interval of the meta-analysis (41.96 to 65.84%), it should be noticed that the meta-analysis included only spinal pain, while we included not only pain but also other symptoms. We also included regions other than the spine. Moreover, subjects in our study were far younger in practice than those in the meta-analysis.

Although the subjects in our study had practised dentistry for less than five years, the prevalence is high. Yi^5^ reported that high and specialty-related musculoskeletal disorders afflict dental professionals even since early training years. Rising^8^ reported body pain in 46 to 71% of dental students with increasing percentage with the years in dental school.

In our study, prolonged sitting was the most common aggravating factor. This is consistent with the finding of Harshid^9^ who reported that most musculoskeletal symptoms in dentists were aggravated by prolonged sitting. In contrast, Harshid^9^ found correct posture was the main relieving factor while we found that exercise was the main relieving factor.

Neck is the most common affected region. This finding is in line with the study of Kierklo^10^. During common dental procedures, upper trapezius muscle was the most exerted muscle^11^. Using electromyographic study, Mileard^12^ also found that trapezius muscle had the highest myoelectric activity.

Our finding is consistent with Bozini^13^ that there is association between occupational stress and musculoskeletal pain. However, it is Bozini^13^ who argued that workers who reported musculoskeletal pain were more likely to develop subsequent perceptions of stress. Accordingly, our data was insufficient to blame stress as the cause of musculoskeletal disorders among dentists.

Our data suggest positive association between exercise and musculoskeletal disorder among dentists. Exercise may help to alleviate musculoskeletal disorders although we did not evaluate the types of exercises the dentists performed to alleviate their symptoms. However, Kumar *et al*^14^ highlighted exercises for prevention of musculoskeletal disorders in dentists.

We also found that body mass index is associated with stiffness, fatigue and discomfort. Possible explanation for this is that higher body mass index increased mechanical demands and metabolic factors. Increased body mass index also increased forces across the joints, especially in weight bearing joints^15^.

## Conclusions

The prevalence of musculoskeletal disorders in young dentists is 63.5%. Neck is the most common affected region. Stress and exercise are the main associated factors for musculoskeletal problems in dentists.
